# Psychosocial working conditions as determinants of concerns to have made important medical errors and possible intermediate factors of this association among medical assistants – a cohort study

**DOI:** 10.1186/s12913-022-08895-2

**Published:** 2022-12-09

**Authors:** Viola Mambrey, Peter Angerer, Adrian Loerbroks

**Affiliations:** Institute of Occupational, Social and Environmental Medicine, Centre for Health and Society, Faculty of Medicine, University of Düsseldorf, Moorenstr. 5, 40225 Düsseldorf, Germany

**Keywords:** Cohort study, Germany, Medical assistant, Medical error, Patient safety, Psychosocial working conditions, Quality of care

## Abstract

**Objective:**

We sought to examine the association of psychosocial working conditions with concerns to have made important medical errors and to identify possible intermediate factors in this relationship.

**Methods:**

We used data from 408 medical assistants (MAs) in Germany who participated in a 4-year prospective cohort study (follow-up period: 03–05/2021). Psychosocial working conditions were assessed at baseline by the effort-reward imbalance questionnaire and by a MA-specific questionnaire with seven subscales. MAs reported at follow-up whether they are concerned to have made an important medical error throughout the last 3 months, 12 months or since baseline (yes/no). These variables were merged into a single variable (any affirmative response vs. none) for primary analyses. Potential intermediate factors measured at baseline included work engagement (i.e., vigor and dedication, assessed by the UWES), work satisfaction (COPSOQ), depression (PHQ-2), anxiety (GAD-2) and self-rated health. We ran Poisson regression models with a log-link function to estimate relative risks (RRs) and 95% confidence intervals (CIs). Doing so, we employed the psychosocial working condition scales as continuous variables (i.e. z-scores) in the primary analyses. Potential intermediate factors were added separately to the regression models.

**Results:**

Poor collaboration was the only working condition, which was significantly predictive of the concern of having made an important medical error (RR = 1.26, 95%CI = 1.00–1.57, *p* = 0.049). Partial intermediate factors in this association were vigor, depression and anxiety.

**Conclusion:**

We found weak and mostly statistically non-significant associations. The only exception was poor collaboration whose association with concerns to have made an important medical error was partially explained by vigor and poor mental health.

**Supplementary Information:**

The online version contains supplementary material available at 10.1186/s12913-022-08895-2.

## Introduction

Psychosocial working conditions in the healthcare sector have been characterized as unfavorable, for instance, in terms of a high workload, frequent interruptions, poor teamwork and unclear work processes [1–4]. Numerous cross-sectional studies among health professions suggest that adverse psychosocial working conditions are associated with potential patient safety issues, such as health professionals’ poor adherence to safety practices and medical errors [5–7]. Patient safety is one the six dimension of quality of care distinguished by the World Health Organization [8]. In addition, unfavorable working conditions increase the risk of poor wellbeing among healthcare personnel [9–11], which in turn, is predictive of adverse patient safety outcomes [12–14]. This renders wellbeing a potential intermediate factor in the association of adverse working conditions and subsequently poor patient safety [15]. The term wellbeing can be conceptualized to encompass both positive psychosocial constructs, such as motivation and satisfaction, and negative constructs, such as depression, anxiety, and poor physical health [16]. While a number of cohort studies have addressed the relationship between wellbeing and quality of care [17, 18], longitudinal research examining the relationship of specifically adverse working conditions with patient safety indicators e.g., medical errors, remains scarce and is limited to the hospital setting [13, 15, 19, 20]. Furthermore, the evidence for this relationship is inconsistent, with some studies finding associations [15, 19, 20] and others not [13]. Identification of the specific unfavorable psychosocial working conditions that are linked to adverse patient safety outcomes may inform early prevention of, for instance, medical errors in contrast to the treatment of the consequences of these conditions in terms of poor wellbeing.

In Germany, about 93% of the population seeks outpatient treatment at least once a year [21]. Among the largest professional groups in outpatient care in Germany are medical assistants (MAs) who support physicians in their daily work [22]. The range of tasks associated with the MA profession is broad and includes administrative duties (e.g., managing practice procedures, accounting, documenting patient histories), but also clinical tasks (e.g., blood sampling, administering injections, wound care, laboratory diagnostics and performing X-rays or electrocardiography) [23]. Also, MAs are usually the first point of contact for patients, as MAs run the reception and answer the phone. Consequently, MAs often need to engage in clinical-decision making by assessing the urgency of patients’ medical complaints. In general practices for instance, severe medical errors (i.e., in terms of potential harm to patients) may mostly result from MAs’ misjudgments when patients contact the practice [24]. Moreover, errors in carrying out diagnostic procedures (e.g., laboratory tests) performed by MAs may lead to misdiagnoses, which carry a high potential for patient harm [25]. Just like members of other health professions, MAs report poor psychosocial working conditions including a high workload, multitasking, poor teamwork and, as consequence, high levels of chronic stress [6, 26]. Further, prior cross-sectional evidence from our group suggests that poor psychosocial working conditions (e.g., collaboration and practice organization) among MAs are associated with reported concerns to have made an important medical error [6].

Prospective studies are needed to clarify the direction of association and potential causality. Thus far, earlier prospective studies on the relationship between adverse psychosocial working conditions and patient safety indicators are sparse, provide inconsistent evidence and to the best of our knowledge such studies are lacking for the primary care setting [13, 15, 19, 20]. Therefore, this study contributes prospective data from a professional group in outpatient care (i.e., MAs) and aims 1) to examine longitudinally if adverse psychosocial working conditions are determinants for concerns to have made an important medical error among MAs and 2) to examine potential intermediate factors (i.e., wellbeing constructs) of this relationship for the first time among MAs in Germany.

## Methods

### Study sample

We drew on data from a cohort study of MAs in Germany. Baseline data were collected between September 2016 and April 2017. The questionnaire was available as an online survey or a hard-copy version. Various associations and organizations supported the nationwide recruitment of participants by distributing flyers, sharing information internally or on their respective homepages and by direct forwarding to relevant institutions. Detailed information on the recruitment efforts is provided elsewhere [6]. MAs currently in training or holding a MA degree were included in the study. In total, 944 MAs completed the baseline questionnaire and the sample was limited to those 887 MAs who reported to be employed as a MA. For the follow-up data collection (March until May 2021), participants were invited to participate via an e-mail or a post letter. The invitation included an individualized link to the online survey. The contacted MAs could also request to receive hard copies of the questionnaire for completion instead. Reminders were sent out 3 weeks and 6 weeks after the initial invitation. In total, 537 MAs (56.89%) participated at follow-up.

For the current longitudinal analysis, we included only participants who reported to be in current employment as a MA at both time points (*n* = 408) (e.g., as opposed to current employment but not as a MA, unemployment, parental leave or retirement, as reported by follow-up participants who were not eligible). In the analytical sample, the mean individual follow-up period was 4.40 years (standard deviation [SD] = 0.10) ranging from 4.04 years to 4.62 years The Ethics Committee of the Medical Faculty of the Heinrich-Heine-University of Düsseldorf approved our study (registration number of the baseline study: 4778 and follow-up study: 2019-819_2).

### Theoretical framework and its operationalization

**Fig. 1:**
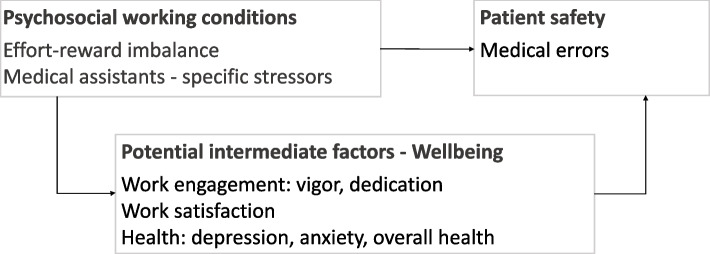
Theoretical framework used in this study. Model adapted from Angerer and Weigl (2015) showing potential pathways between psychosocial working conditions measured at baseline and patient safety assessed at follow-up with potential intermediate factors at baseline among MA

The model used in this study is based on a theoretical framework proposed for the physician profession [2, 5]. The framework postulates two pathways: the direct pathway assumes that psychosocial working conditions will exert themselves effects on the quality of care. The indirect pathway hypothesizes that psychosocial working conditions affect wellbeing, which acts as an intermediate factor as it in turn impacts the quality of care [5]. We adapted the theoretical framework for our study and show the concept used to operationalize each overarching component in Fig. 1.

### Questionnaires

#### Determinants: psychosocial working conditions at baseline

The established effort-reward imbalance (ERI) questionnaire [27] and a questionnaire capturing MA-specific working conditions [6] were used to measure psychosocial working conditions. The ERI questionnaire was administered at baseline and comprises 17 items measuring the sub-dimensions effort [6 items, i.e., high workload, time pressure and responsibility] and reward [11 items, i.e., high salary, high esteem and good career prospects]. Items are presented as statements and the level of agreement is expressed using a 4-point Likert scale ranging from “I strongly disagree” (1) to “I strongly agree” (4). The higher the score, the higher the level of agreement with the respective subdimension. The ERI model postulates that an imbalance between high effort spent and low reward received causes work-related distress. The degree of imbalance at the individual level is represented by the ERI ratio, with an ERI ratio value > 1.0 indicating work stress. The ERI ratio is created based on the sum scores of the subdimensions effort [6 items, potential score range = 6 to 24] and reward [11 items, potential score range = 11 to 44] weighted by the opposite number of items.

The MA-specific questionnaire was developed by our group based on prior qualitative research [1], was refined by cognitive interviews and psychometrically evaluated [6]. The questionnaire consists of 29 items that are presented as statements and responses are provided on a 4 point Likert scale varying from “I strongly disagree” (1) to “I strongly agree” (4). Factor analyses grouped those items into 7 types of psychosocial working conditions comprising each 3–6 items [6]. We calculated factor-specific sum scores while reversing some items to harmonize the interpretation of answers [6]. With an increase in the score, the exposure to the respective stressor increases. The 7 factors are: (1) workload [e.g., time pressure, high number of patients; 6 items; potential score range = 6–24], (2) job control [e.g., documentation effort, interruptions and multitasking; 6 items; potential score range = 6–24], (3) collaboration with supervisor/colleagues [e.g., working climate; 4 items; potential score range = 4–16], (4) gratification [e.g., career prospects and recognition; 4 items; potential score range = 4–16], (5) practice organization [e.g., work structure and responsibilities; 3 items; potential score range = 3–12], (6) resources [e.g., interaction with patients and variety of work tasks; 3 items; potential score range = 3–12], (7) leadership behavior [e.g., recognition and work organization; 3 items; potential score range = 3–12].

#### Potential intermediate factors: wellbeing at baseline

Some factors may play an intermediary role with regard to the link between psychosocial working conditions and the concerns to have made important medical errors. Specifically, such intermediate factors may explain those associations, because they are a potential consequence of psychosocial working conditions and may in turn contribute to concerns related to important medical errors [5]. We considered the following potential intermediate factors:

1. Work engagement describes a positive and motivational mindset concerning work [28]. Work engagement can be measured by the 9-item Utrecht Work Engagement Scale (UWES) with the three dimensions: vigor [e.g., high work energy, work-related persistence; 3 items], dedication [e.g., inspiration, pride in work; 3 items] and absorption [e.g., feeling completely absorbed in one’s work; 3 items] [28]. In the current study, assessment of work engagement was limited to the two subscales vigor and dedication because previous studies suggest that vigor and dedication, specifically, are determinants of quality of care rather than absorption [29, 30]. Responses are given on a 7-point Likert scale indicating frequency from “never” (0) up to “always” (6). Sum scores were calculated and divided by the respective number of items with a potential score range between 0 and 6.
2. Work satisfaction was measured by a single item from the first version of the Copenhagen Psychosocial Questionnaire (COPSOQ) [31, 32] (“Regarding your work in general. How pleased are you with your job as a whole, everything taken into consideration?”). Responses are provided on a 4-point Likert scale varying between “very unsatisfied” (0) and “very satisfied” (3).
3. Health variables included depressive symptoms and anxiety and overall health status. Depressive symptoms and anxiety were assessed by the patient health questionnaire (PHQ-2) and the generalized anxiety disorder questionnaire (GAD-2), respectively [33]. The items are presented as statements and answered on a 4-point Likert scale inquiring after the frequency of symptoms ranging from “not at all” (0) to “almost every day” (3). For each instrument the score can range from 0 to 6. Overall health status (self-rated health) was assessed by the item “How is your health status in general?” using a 5-point Likert scale for responses (very good (1), good (2), average (3), bad (4), very bad (5)) [6, 34].

#### Outcomes: concern to have made an important medical error at follow-up

At follow-up, participants reported based on three separate items whether they are concerned to have made an important medical error in the last 3 months [no/yes], in the last 12 months [no/yes] or since baseline [no/yes]. In order to maximize the sensitivity to detect any error, perceived concerns of having made an important medical error in the past 3 months, 12 months or since baseline were merged into a single variable called “summary measure of important medical errors” (any affirmative response vs. none). We elaborate on the strengths and weaknesses of our approach to measuring errors in the discussion section.

### Statistical analysis

The primary statistical analysis was based on z-standardized continuous exposure variables (e.g., psychosocial working conditions) and the dichotomous outcome variable (i.e., summary measure of important medical errors). We ran Poisson regression models with robust estimators and a log-link function to estimate relative risk (RR) and corresponding 95% confidence intervals (CIs) [35]. For each working condition exposure (i.e., effort, reward, ERI ratio and the seven MA-specific working condition subscales) a separate Poisson regression model was computed. First, we ran unadjusted models and then adjusted the models for age and leadership position [30]. Originally, we intended to control for sex as a confounding factor. Due to a very low number of non-female participants (*n* = 5, 1.23%) and thus highly unlikely confounding analyses we decided not to adjust for sex.

In addition, we estimated the RR of the association between exposure and outcome adjusted for the potential intermediate wellbeing constructs by adding the potential intermediate factors separately as continuous variables (i.e., vigor, dedication, depression, and anxiety [z-scores]) or ordinal variables (i.e., work satisfaction and self-rated health) to the regression models. An attenuation of the association between exposure and outcome adjusted for the intermediate factor towards the null value of RR = 1.0 was considered to suggest mediation [36].

We ran various types of sensitivity analysis to explore the robustness of our findings. Firstly, we re-ran the primary analysis using dichotomized psychosocial working condition exposures instead of the continuous exposures. In those analyses, dichotomization of the ERI ratio was based on the theory-based cut-off (> 1.0 vs ≤1.0). In accordance with previous research [4, 6, 37] the remaining exposures were dichotomized based on their distribution, with the cut-off being set at the highest tertile versus the remaining tertiles. Secondly, we repeated our primary analyses using the outcome variables separately and thus for different reference periods (i.e., concerns to have made an important medical error in the last 3 month, the last 12 months or since baseline, respectively).

We also ran two types of non-responder analyses based on all variables included in the current analyses. First, we compared the baseline characteristics of those baseline participants that were employed as MAs at baseline and that participated at follow-up (*n* = 507) versus those who did not (*n* = 380) using *chi-square* tests for nominal (e.g., sex, employment status) and ordinal variables (e.g., work satisfaction, self-rated health) and *Student’s t-*test for continuous variables (e.g., ERI ratio, subscale factors of MA-specific questionnaire, vigor), respectively. In addition, we ran Poisson regressions with separate models for each baseline variable and a model including all variables to predict the likelihood of participation at follow-up expressed as RRs and 95% CIs.

For the statistical analysis IBM SPSS 25.0 was used. Missing values were not imputed and the range of percentage of missing values was 0.00% (i.e., errors, vigor and work satisfaction) to 6.86% (i.e., ERI ratio).

## Results

### Non-responder analyses and characteristics of participants

Follow-up participants differed from non-participants in terms of some socioeconomic characteristics and some health variables (see supplementary material table A1): Follow-up participants were older than non-participants (41.86 vs. 35.82 years, *p* < 0.001) and consequently had more years of work experience (19.34 vs. 14.32 years, *p* < 0.001). Further, follow-up participants were less likely to work full time than non-participants (54.73 vs. 65.38%, *p* = 0.002). Moreover, with regard to depressive symptoms, follow-up participants showed slightly lower mean values on the PHQ-2 than non-participants (mean values: 1.47 vs. 1.67, *p* = 0.046). There was no evidence for differences regarding the psychosocial working conditions though. The Poisson regression analysis with separate models for each variable confirmed the results of the above-mentioned non-responder analysis, with the variables age (continuous), work experience (continuous), and employment status (full-time vs. part time) being predictive of participation (RR = 1.02, 95%CI = 1.01–1.03; RR = 1.02, 95%CI = 1.01–1.02 and RR = 1.20, 95%CI = 1.07–1.35, respectively). In the Poisson regression model, which included all variables (i.e., demographic variables, work-related variables, exposures and intermediate factors), only age was a significant predictor of participation (RR = 1.02, 95%CI = 1.01–1.03) (data not shown).

In total, 98.77% of the MAs were female with a mean age of 41.81 years (SD = 10.38 years, see Table 1). In terms of occupational characteristics, 53.75% of the MAs worked full time and 50.25% reported to hold a leadership position. In total, 71.84% of the MAs reported work stress according to the ERI ratio. Based on the potential score range of each respective exposure variable, effort, low job control and poor leadership behavior seemed to be particularly pronounced. The outcome summary measure of concerns to have made an important medical error yielded a prevalence of 11.03% (*n* = 45). The percentages of the remaining outcomes were 5.39% (*n* = 22) for concerns to have made an important medical error in the last 3 month, 6.13% (*n* = 25) for the last 12 months and 8.09% (*n* = 33) for concern to have made an error since baseline.

**Table 1:** Characteristics of the study population (*n* = 408*)

**Characteristics**		
Age, mean (M), standard deviation (SD)	41.81	(10.38)
Work experience (in years), M, (SD)	19.36	(10.96)
	***n***	**(%)**
Sex		
Male	5	(1.23)
Female	401	(98.77)
Employment status		
Full time	215	(53.75)
Part time	185	(46.25)
Leadership position		
Yes	204	(50.25)
Work stress according to ERI^a^ (i.e., ratio > 1.0)		
Yes	273	(71.84)
	**M**	**(SD)**
Effort	18.50	(3.17)
Potential range 6–24		
Reward	28.68	(6.04)
Potential range 11–44		
ERI ratio^a^	1.25	(0.41)
MA^b^ sub-scale (high) workload	17.31	(4.30)
Potential range 6–24		
MA sub-scale (low) job control	21.17	(2.75)
Potential range 6–24		
MA sub-scale (poor) collaboration	8.20	(2.82)
Potential range 4–16		
MA sub-scale (low) gratification	11.38	(2.74)
Potential range 4–16		
MA sub-scale (poor) practice organization	6.52	(2.06)
Potential range 3–12		
MA sub-scale (lack of) resources	4.55	(1.64)
Potential range 3–12		
MA sub-scale (poor) leadership behavior	7.99	(2.29)
Potential range 3–12		
Vigor^c^	3.52	(1.34)
Value range 0–6		
Dedication^c^	3.86	(1.38)
Value range 0–6		
Depression^d^	1.39	(1.43)
Value range 0–6		
Anxiety^e^	1.36	(1.62)
Value range 0–6		
	***n***	**(%)**
Work satisfaction^f^		
Very unsatisfied	10	(2.45)
Unsatisfied	89	(21.81)
Satisfied	262	(64.22)
Very satisfied	47	(11.52)
Self-rated health		
Very good	79	(19.65)
Good	174	(43.28)
Average	126	(31.34)
Poor	20	(4.98)
Very poor	3	(0.75)
Important medical error^g^		
Last 3 months	22	(5.39)
Last 12 months	25	(6.13)
Since baseline	33	(8.09)
Summary measure of	45	(11.03)
important medical errors		

*n with complete data on the respective variable and item

^a^effort-reward imbalance questionnaire (ERI) ERI = (Effort*11)/(Reward*6)

^b^medical assistant (MA)

^c^sub-dimension of Utrecht Work Engagement Scale

^d^Patient Health Questionnaire (PHQ-2)

^e^Generalized Anxiety Disorder questionnaire (GAD-2)

^f^Copenhagen Psychosocial Questionnaire

^g^perceived concerns about having made an important medical error reported for the last three months, 12 months and since baseline merged into a single variable (any affirmative response vs none)

### Primary statistical analysis

The primary statistical analyses (see Table 2) showed that the magnitude of the examined associations between adverse working conditions (z-score) and the summary measure of important medical errors ranged from moderate to lacking and were overall statistically non-significant. Poor collaboration was the only type of working condition, which was significantly predictive of the concerns of having made important medical errors, that is, an increase in one SD in poor collaboration (SD = 2.82, see Table 1) increased the risk of concern by 26% to have made a summary measure of important medical error (RR = 1.26; 95%CI = 1.00–1.57). Potential weak positive associations with the outcomes, which were non-significant though, were observed for high workload and poor practice organization (RR = 1.18, 95%CI = 0.93–1.51 and RR = 1.15, 95%CI = 0.89–1.48, respectively).

**Table 2:** Risk of being concerned to have made an important medical error across the full follow up period (summary measure variable*) by exposure to adverse psychosocial working conditions at baseline (Poisson regression)

		Summary measure of concerns to have made an important medical error			
Characteristic		Model I^**a**^		Model II^**b**^	
		RR^**c**^	95% CI^**d**^	RR^**c**^	95% CI^**d**^
ERI model					
Effort	z-score^e^	0.94	0.74, 1.20	0.98	0.77, 1.25
Reward	z-score	0.80	0.63, 1.03	0.86	0.66, 1.12
ERI ratio	z-score	1.09	0.84, 1.40	1.06	0.82, 1.36
MA-specific instrument					
Workload	z-score	1.16	0.91, 1.47	1.18	0.93, 1.51
Job control	z-score	0.94	0.74, 1.18	1.00	0.78, 1.27
Collaboration	z-score	1.31	1.04, 1.64	1.26	1.00**, 1.57
Gratification	z-score	1.11	0.86, 1.43	1.03	0.79, 1.35
Practice organization	z-score	1.21	0.95, 1.55	1.15	0.89, 1.48
Resources	z-score	0.81	0.59, 1.12	0.80	0.58, 1.10
Leadership behavior	z-score	1.14	0.85, 1.53	1.10	0.82, 1.48

* perceived concerns about having made an important medical error reported for the last three months, 12 months and since baseline merged into a single variable (any affirmative response vs none); effort-reward imbalance questionnaire (ERI) or medical assistant (MA)-specific work stress questionnaire

^a^unadjusted

^b^additionally adjusted for age and leadership position

^c^relative risk (RR) and

^d^95% confidence intervals (95% CIs)

^e^ a higher score reflects higher agreement to the stressor

** exact *p*-value = 0.04953

In mediation analysis, we observed that the association between poor collaboration and reporting to be concerned to have made a summary measure of important medical error (RR = 1.26) was attenuated toward the null value by the intermediate factors vigor (RR_adjusted_ = 1.16), depression (RR_adjusted_ = 1.09) and anxiety (RR_adjusted_ = 1.06) (see supplementary material Tables A2a and b). For the sake of completeness, we reported all estimates of the association between adverse working conditions and the summary measure of important medical errors outcome adjusted for the potential intermediate factor (see supplementary material Table A2a and b). However, those results seem to have little relevance as associations between adverse working conditions (except for collaboration) and the summary measure of important medical errors were not significant in the primary analysis.

### Sensitivity analyses

The sensitivity analyses based on dichotomized adverse working conditions suggested similar associations as the primary analyses (supplementary material Table A3.). In detail, we observed a significant, yet potentially random inverse association between high versus low reward and the summary measure of important medical errors (RR = 0.46, 95%CI = 0.21–0.98). Strong positive associations with the outcome, though non-significant, were found for the dichotomized poor collaboration exposure variable (RR = 1.54, 95%CI = 0.89–2.67) and the dichotomized poor practice organization variable (RR = 1.58, 95%CI = 0.88–2.83). We also found similar associations when we employed the three outcome variables reflecting concerns to have made an important medical error within the last 3 months, last 12 months and since baseline separately (supplementary material table A4.-A6.).

## Discussion

Overall, we found rather weak and statistically non-significant associations between adverse psychosocial working conditions and subsequent concerns to have made an important medical error. Poor collaboration was found to be moderately predictive of the concern to have made an important medical error across all time periods examined. This association was mediated by the potential intermediate factors vigor, depression, and anxiety. In addition, a potential trend towards positive associations was observed for higher workload and poorer practice organization with the outcome.

### Comparison to prior research

The few earlier prospective epidemiological studies partially found links between psychosocial working conditions and patient safety [13, 15, 19, 20]. The comparison with the existing literature is limited though as the specific combination of exposure and outcome constructs used in this study of unfavorable psychosocial working conditions with patient safety in terms of medical errors has only been applied in one prospective study [15]: That study – carried out among nurses in Japan – documented a link between several job stressors (i.e., nursing stress scale e.g., conflicts with supervisor or colleagues, high workload, lack of support), and the subsequent self-reported frequency of near misses and adverse events combined into a medical error risks variable [15]. However, those job stressors were combined into a single variable thereby limiting comparability with the specific work stressors and resources (e.g., collaboration, workload) analyzed in our study. Therefore, the definitions of psychosocial working conditions and patient safety in terms of medical errors were broadened to include safety culture [13] and other indicators of the quality of care or overall patient safety [19, 20] to draw on further prospective studies: A study among hospital physicians in Germany suggested associations of high social stressors (e.g., conflicts with colleagues and supervisors) and time pressure with the physicians’ perception of the impairment of the quality of care they provide due to working conditions [19]. A study from Switzerland analyzed and confirmed the link between teamwork and clinician-rated overall patient safety [20]. By contrast, a study among hospital staff in France that used observer-based ratings to assess medical errors (e.g., error of execution or error of planning) and adverse events found only weak and statistically non-significant links between safety culture and medical errors [13]. Our study adds to the sparse existing literature by providing the first prospective evidence for MAs and thus for health care staff that is mainly employed in the outpatient care sector.

Prior cross-sectional evidence from our study suggested that the ERI components (i.e., high effort, high reward, high ERI ratio) as well as poor collaboration and poor practice organization are strongly and significantly associated with the concerns to have made an important medical error in the last 3 months among MAs [6]. The results from our current prospective study may support those earlier cross-sectional findings in some instances, i.e., by highlighting poor collaboration and possibly poor practice organization to be predictive of the concern to have made an important medical error among MAs. In our study, the construct collaboration is defined by interpersonal relationships (e.g., conflicts with colleagues or supervisors, unfair treatment), while the processes of cooperation are covered by the factor practice organization (e.g., well-structured work processes, responsibilities). Our results illustrate a rather moderate relationship of poorer collaboration with the concern to have made an important medical error. The notion of a relationship of collaboration with patient safety is in line with the abovementioned prospective studies [15, 19, 20]: Whereas the study among hospital physicians in Germany found that pronounced social stressors (e.g., conflicting relationship with colleagues and supervisors) are directly related to a lower self-rated quality of care [19], the study from Switzerland focusing on the interplay between different types of teamwork and physicians’ rated overall patient safety in intensive care units (ICU) found that interpersonal teamwork (i.e., equivalent to collaboration as measured in this study) predicted patient safety only indirectly, that is, through team organizational and coordination behaviors [20]. The authors of the latter study argued that positive interpersonal teamwork may enhance communication within the team, which in turn increases patient safety. This hypothesis is supported by the longitudinal study among nurses in Japan which found a lack of communication to be associated with job stressors including collaboration, which in turn were associated with the risk of medical errors [15]. Therefore, communication may potentially explain the link between collaboration and patient safety observed in our study. In addition, we found that poor mental health (i.e., depression and anxiety) and vigor, may partially explain the observed positive relationships between poor collaboration and reported concerns to have made important medical errors. Poor mental health may develop due to poor collaboration [10] and may in turn contribute to subsequent medical errors [13, 38]. This is in line with the study by Tanaka et al. (2012) which found depression to be an intermediate factor between job stressors and perceived risk of medical errors. Further, one may speculate that communication is another factor on that pathway as poor mental health is often associated with difficulties in social interactions and thus likely also with poor collaboration [39]. In terms of positive wellbeing that can emerge from favorable working conditions, good collaboration may lead to work engagement in the form of vigor [40]. The high work energy and work-related persistence associated with vigor may in turn reduce the risk of medical errors [29, 30]. Our study may illustrate that poor collaboration contributes to worse patient safety and that this relationship may be partially mediated by depression, anxiety and vigor.

In our study, we observed a potential pattern of positive associations of higher workload and poorer practice organization with an increase in reported concerns to have made an important medical error. The longitudinal study among hospital physicians in Germany found that time pressure was linked to a diminished physician-rated quality of care [19]. Time pressure, captured by the factor “workload” in this study, was assumed to be a structural deficiency within the care system that prevents health staff from performing their tasks effectively [19]. An observer-based study among ICU health staff in France found that high workload increased the risk of medical errors by almost 50% [13]. A high workload in combination with suboptimal staff planning was considered part of the organizational factors hypothesized to increase medical errors [13]. Inefficient practice organization has been reported to add to the burden of workload in a qualitative study among MAs [41]. Our results indicate that poorer practice organization may potentially be an independent determinant of reported concerns to have made an important medical error. A cross-sectional study among physicians in primary care from the US measured the perception of the atmosphere at the workplace (i.e., ranging from calm to hectic or chaotic) and found that chaotic workplaces were associated with higher rates of medical errors [42]. An observer-based study among MAs in Germany found that well-structured tasks and a clear responsibility was relevant for a functioning workflow, as MAs’ work processes were frequently interrupted [43]. Interruption in turn may lead to a reduced patient safety in terms of medical errors [44]. Overall, poor practice organization and high workload are processes and structural determinants that may facilitate the occurrence of errors [45]. It needs to be mentioned again though that the corresponding associations were weak and non-significant in our study. We hope however that our discussion stimulates more research into those potentials determinants of patient safety.

### Methodological considerations

A strength of this study is its prospective design. Another strength is our comprehensive assessment of psychosocial working conditions of MAs, which relied on both an established generic instrument (e.g., the ERI model) and an instrument specifically developed for MAs.

While these two instruments represent a broad spectrum of psychosocial working conditions that are key working conditions according to MAs [1], further relevant work stress models such as the job demand-control model or organizational justice [46, 47] may provide additional insights into the psychosocial working conditions that predict errors [48, 49]. Unfortunately these concepts were not included in this study.

A limitation of this study is the rather small sample size for prospective analyses. This may have restricted the statistical power, which limits the detection of statistically significant associations. The links of collaboration and the pattern of positive association of workload and practice organization with the outcome may have been random findings due to multiple testing. After Bonferroni correction those estimates were not significant anymore. The frequency of reported concerns to have made an important medical error was low and thus yielded a limited number of cases (i.e., only 45 cases for our primary outcome variable). This limits the feasibility to adjust for a large set of confounders; however we needed to adjust for two confounders only (i.e., age and leadership position).

Selection bias cannot be ruled out. However, firstly, thorough non-responder analysis (see supplementary material Table A1) did not yield significant differences regarding exposure variables (e.g., psychosocial working conditions). Secondly, our study sample is fairly representative of the German MA population according to the Federal Statistical Office of Germany in terms of sex, age, employment status [22] as well as comparable in terms of age, work experience, marital status to a previous study among MAs in Germany that claims to be representative [26].

Our study relied on longitudinal assessments at two time points. However, a three-wave study had been superior to analyze the intermediary position of potential intermediate factors between the relationship of psychosocial working conditions and patient safety, as intermediate factors e.g., depression could also be conceptualized as a shared cause of poor collaboration and medical errors [14, 39, 50].

A further limitation, which could introduce information bias is that we measured patient safety by self-reported “*concerns* to have made an *important medical error*”. Participants were not given a definition of an *important* medical error. Therefore, the understanding of what an important error constitutes may have differed across MAs. As a consequence, some MAs may have subjectively considered some errors as unimportant and accordingly did no report them. In addition, important errors may have been made, but immediately corrected by the MA or a supervisor and therefore are not recalled as errors. This might have reduced the likelihood to report errors. Minimization of such potential under-reporting bias might not only have improved the validity of our findings, but also the precision of estimations due to a higher number of reported errors. The approach to assess “important medical errors” allowed us to obtain information on all types of errors rather than one specific type of error. In addition, errors were assessed by *concerns* and not perceived actual error frequency. Reasons for concealing errors are attributed to fear of individual accountability, judgement of capability, and legal consequences [51]. The approach to measure *concerns*, which has been applied in further studies [12], therefore may have the advantage of lowering the threshold to report errors as participants feel less exposed to social desirability and possible legal consequences. Nevertheless, pronounced c*oncerns* may occur as a symptom of depression and anxiety. Therefore, we ran additional analysis excluding participants with poor mental health (i.e., depression or anxiety) from the primary analysis to rule out confounding. The estimates of the primary analysis were not altered though (data not shown).

In addition, it should be emphasized that many wellbeing concepts - e.g., depression and overall health - can deteriorate independently from work and therefore cannot be conceptualized per se as work-related. We therefore recommend to additionally examine burnout in future research as the development of this syndrome is closely tied to the workplace, which unfortunately was not feasible in this study.

### Recommendations for future research and the practice

Further studies are needed to support our findings. Those studies should be prospective and focus further on working conditions as a potential starting point rather than wellbeing as it might act as an intermediate factor [15]. By identifying the adverse working conditions associated with perceived medical errors, it is possible to intervene at the core structures and processes of medical errors rather than treating the consequences of these deficiencies in terms of impaired wellbeing. Moreover, wellbeing should be analyzed as a potential intermediate factor in greater depth, with a stronger emphasis on salutogenic wellbeing constructs (e.g., work engagement). So far, only pathogenic wellbeing constructs have been tested longitudinally for mediation of the relationship of working conditions with patient safety [15, 19, 20].

Although the significance of our findings still needs to be substantiated by further prospective studies, our study suggests that poor collaboration may be a promising starting point in order to address patient safety in outpatient care. In practice, this could be addressed through regular team meetings of the entire team as well as solely among the MAs, constructive feedback sessions with the supervising physician(s) to strengthen communication, and involvement of MAs in staff-related decisions and team activities [52, 53]. As structural processes and delegation of tasks in the workplace are factors contributing to patient safety [54], interventions should focus on practice organization and workload by efficiently structuring the daily practice routine, e.g., minimizing patients’ stay at the reception to reduce the likelihood of concurrent stressors, relocating answering of the telephone to a separate room and clearly assign work tasks and responsibilities [52, 53]. Finally, the implementation of error management systems in outpatient care practices is needed to strengthen error reporting and establish clear responsibilities within the team for error management, which could promote communication about errors and subsequent lead to higher patient safety [55].

## Conclusion

Our study is the first to prospectively examine the relationship between a broad range of distinctive psychosocial working conditions and concerns to have made important medical errors among MAs in Germany. Overall, associations were mostly non-significant. We found though that collaboration and potentially also workload and practice organization may be predictive of reported concerns to have made an important medical error. The few potentially meaningful associations that were observed were partially mediated by vigor and poor mental health.

## Supplementary Information

**Additional file 1: Table A1.** Comparison of baseline characteristics of follow-up participants (n*=507) compared to follow-up non-participants (n*=380). **Table A2a.** Risk of being concerned to have made an important medical error since baseline for the follow up period combined by exposure to adverse psychosocial working conditions at baseline including potential mediators (Poisson regression). **Table A2b.** Risk of being concerned to have made an important medical error since baseline for the follow up period combined by exposure to adverse psychosocial working conditions at baseline including the mediators (Poisson regression). **Table A3.** Risk of being concerned to have made an important medical error across the full follow up period (summary measure variable*) by exposure to adverse psychosocial working conditions (dichotomized) at baseline (Poisson regression). **Table A4.** Risk of being concerned to have made an important medical error across the last 3 months at follow-up by exposure to adverse psychosocial working conditions at baseline (Poisson regression). **Table A5.** Risk of being concerned to have made an important medical error across the last 12 months at follow-up by exposure to adverse psychosocial working conditions at baseline (Poisson regression). **Table A6.** Risk of being concerned to have made an important medical error since baseline by exposure to adverse psychosocial working conditions at baseline (Poisson regression).

## Data Availability

The datasets generated and analyzed during the current study are not publicly available due to privacy concern, but are available from the corresponding author upon reasonable request.

## Abbreviations

CI: Confidence interval
COPSOQ: Copenhagen psychosocial questionnaire
GAD: Generalized anxiety questionnaire
ERI: Effort reward imbalance
ICU: Intensive care unit
MA: Medical assistant
PHQ: Patient health questionnaire
RR: Relative risk
SD: Standard deviation
UWES: Utrecht work engagement scale
