# Supplementary material for: Psychosocial working conditions as determinants of concerns to have made important medical errors and possible intermediate factors of this association among medical assistants – a cohort study
Source: BMC Health Serv Res. 2022 Dec 9;22:1501. doi: 10.1186/s12913-022-08895-2 (PMC9733172; doi:10.1186/s12913-022-08895-2)
Supplement: Supplementary file 1 — Additional file 1: Table A1. Comparison of baseline characteristics of follow-up participants (n*=507) compared to follow-up non-participants (n*=380). Table A2a. Risk of being concerned to have made an important medical error since baseline for the follow up period combined by exposure to adverse psychosocial working conditions at baseline including potential mediators (Poisson regression). Table A2b. Risk of being concerned to have made an important medical error since baseline for the follow up period combined by exposure to adverse psychosocial working conditions at baseline including the mediators (Poisson regression). Table A3. Risk of being concerned to have made an important medical error across the full follow up period (summary measure variable*) by exposure to adverse psychosocial working conditions (dichotomized) at baseline (Poisson regression). Table A4. Risk of being concerned to have made an important medical error across the last 3 months at follow-up by exposure to adverse psychosocial working conditions at baseline (Poisson regression). Table A5. Risk of being concerned to have made an important medical error across the last 12 months at follow-up by exposure to adverse psychosocial working conditions at baseline (Poisson regression). Table A6. Risk of being concerned to have made an important medical error since baseline by exposure to adverse psychosocial working conditions at baseline (Poisson regression). [file 12913_2022_8895_MOESM1_ESM.docx]

Table A1. Comparison of baseline characteristics of follow-up participants (*n**=507) compared to follow-up non-participants (*n**=380)

| **Characteristics at baseline** |  | **Follow-up participants** | | **Follow-up**  **non-participants** | |  |
| --- | --- | --- | --- | --- | --- | --- |
|  |  | **Mean (M)** | **Standard deviation (SD)** | **M** | **(SD)** | ***t*-test**  **p-value** |
| Age |  | 41.86 | (10.96) | 35.82 | (11.15) | 0.000^+^ |
| Work experience | Years | 19.34 | (11.31) | 14.32 | (10.54) | 0.000^+^ |
|  |  | ***n*** | **(%)** |  |  | ***Chi square* test**  **p-value** |
| Sex | Male | 8 | (1.59) | 6 | (1.60) | 0.988 |
|  | Female | 496 | (98.41) | 369 | (98.40) |  |
| Employment status | Full time | 272 | (54.73) | 238 | (65.38) | 0.002^+^ |
|  | Part time | 225 | (45.27) | 126 | (34.62) |  |
| Leadership position | No | 256 | (50.69) | 200 | (53.76) | 0.368 |
|  | Yes | 249 | (49.31) | 172 | (46.24) |  |
| Work stress according to  ERI ^a^ (i.e,. ratio >1.0) | No  Yes | 121  354 | (25.47)  (74.53) | 98  262 | (27.22)  (72.78) | 0.569 |
| **Exposures** |  | **M** | **(SD)** | **M** | **(SD)** | ***t*-test**  **p-value** |
| Effort |  | 18.63 | (3.20) | 18.47 | (3.18) | 0.456 |
| Reward |  | 28.26 | (5.91) | 28.24 | (6.08) | 0.961 |
| ERI ratio ^a^ |  | 1.28 | (0.41) | 1.28 | (0.44) | 0.861 |
| MA^b^ sub-scale (high) workload |  | 17.52 | (4.26) | 17.15 | (4.10) | 0.199 |
| MA sub-scale (low) job control |  | 21.24 | (2.72) | 20.94 | (2.71) | 0.102 |
| MA sub-scale (poor) collaboration |  | 8.39 | (2.84) | 8.44 | (2.87) | 0.781 |
| MA sub-scale (low) gratification |  | 11.52 | (2.67) | 11.51 | (2.65) | 0.977 |
| MA sub-scale (poor) practice organization |  | 6.58 | (2.06) | 6.53 | (2.10) | 0.731 |
| MA sub-scale (lack of) resources |  | 4.62 | (1.73) | 4.66 | (1.69) | 0.714 |
| MA sub-scale (poor) leadership behavior |  | 8.12 | (2.30) | 7.85 | (2.42) | 0.092 |

Analyses were limited to baseline participants who reported to be employed as medical assistants at baseline (n=887); *n with complete data on the respective variable and item; Student’s t-test testing mean of variables for significant differences between responders and non-responders; *Chi square* test testing differences between nominal and categorical variables by response and non-response; ^+^significant p-value < 0.05;  ^a^ Effort-reward imbalance questionnaire (ERI); ^b^ Medical assistant (MA); ^c^ sub-dimension of Utrecht Work Engagement Scale; ^d^ Patient Health Questionnaire (PHQ-2); ^e^ Generalized anxiety disorder questionnaire (GAD-2); ^f^ Copenhagen Psychosocial Questionnaire.

| **Mediators** |  |  | **M** | **(SD)** | **M** | **(SD)** | ***t*-test**  **p-value** |
| --- | --- | --- | --- | --- | --- | --- | --- |
| Vigor ^c^ | |  | 3.47 | (1.34) | 3.33 | (1.28) | 0.121 |
| Dedication ^c^ | |  | 3.81 | (1.38) | 3.81 | (1.37) | 0.956 |
| Depression ^d^ | |  | 1.47 | (1.42) | 1.67 | (1.49) | 0.046^+^ |
| Anxiety ^e^ | |  | 1.45 | (1.65) | 1.50 | (1.66) | 0.685 |
|  | |  | ***n*** | **(%)** |  |  | ***Chi square* test**  **p-value** |
| Self rated health | | Very good | 94 | (18.80) | 53 | (14.13) | 0.403 |
|  | | Good | 218 | (43.60) | 172 | (45.87) |  |
|  | | average | 158 | (31.60) | 127 | (33.87) |  |
|  | | Poor | 26 | (5.20) | 18 | (4.80) |  |
|  | | Very poor | 4 | (0.80) | 5 | (1.33) |  |
| Work satisfaction ^f^ | | Very unsatisfied | 13 | (2.56) | 13 | (3.44) | 0.481 |
|  | | unsatisfied | 121 | (23.87) | 82 | (21.69) |  |
|  | | Satisfied | 321 | (63.31) | 234 | (61.90) |  |
|  | | Very satisfied | 52 | (10.26) | 49 | (12.96) |  |

Table A1. Continued.

Analyses were limited to baseline participants who reported to be employed as medical assistants at baseline (n=887); *n with complete data on the respective variable and item; Student’s t-test testing mean of variables for significant differences between responders and non-responders; *Chi square* test testing differences between nominal and categorical variables by response and non-response; ^+^significant p-value < 0.05;  ^a^ Effort-reward imbalance questionnaire (ERI); ^b^ Medical assistant (MA); ^c^ sub-dimension of Utrecht Work Engagement Scale; ^d^ Patient Health Questionnaire (PHQ-2); ^e^ Generalized anxiety disorder questionnaire (GAD-2); ^f^ Copenhagen Psychosocial Questionnaire.

Table A2a. Risk of being concerned to have made an important medical error since baseline for the follow up period combined by exposure to adverse psychosocial working conditions at baseline including potential mediators (Poisson regression).

|  |  | **Summary measure of important medical errors** | | | | | | | | | | | | | | | |
| --- | --- | --- | --- | --- | --- | --- | --- | --- | --- | --- | --- | --- | --- | --- | --- | --- | --- |
| **Characteristic** |  | **Model II ^a^** | | |  | **Vigor^b^** | | |  | **Dedication^c^** | | |  | **Self-rated health^d^** | | | |
|  |  | **RR^e^** | **95% CI^f^** | |  | **RR** | **95% CI** | |  | **RR** | **95% CI** | |  | **RR** | **95% CI** | |  |
| ERI model |  |  |  | |  |  |  | |  |  |  | |  |  |  | |  |
| Effort | z-score | 0.98 | 0.77, | 1.25 |  | 0.92 | 0.70, | 1.21 |  | 0.98 | 0.76, | 1.27 |  | 0.92 | 0.71, | 1.19 |  |
| Reward | z-score | 0.86 | 0.66, | 1.12 |  | 0.95 | 0.70, | 1.30 |  | 0.91 | 0.66, | 1.25 |  | 0.89 | 0.64, | 1.22 |  |
| ERI | z-score | 1.06 | 0.82, | 1.36 |  | 0.95 | 0.70, | 1.30 |  | 1.01 | 0.75, | 1.36 |  | 0.99 | 0.72, | 1.36 |  |
| MA-specific instrument |  |  |  |  |  |  |  |  |  |  |  |  |  |  |  |  |  |
| Workload | z-score | 1.18 | 0.93, | 1.51 |  | 1.14 | 0.87, | 1.48 |  | 1.21 | 0.94, | 1.56 |  | 1.12 | 0.86, | 1.46 |  |
| Job control | z-score | 1.00 | 0.78, | 1.27 |  | 0.96 | 0.74, | 1.24 |  | 1.02 | 0.79, | 1.33 |  | 0.93 | 0.73, | 1.20 |  |
| **Collaboration^g^** | z-score | 1.26 | 1.00, | 1.57 |  | 1.16 | 0.88, | 1.53 |  | 1.22 | 0.95, | 1.57 |  | 1.22 | 0.92, | 1.62 |  |
| Gratification | z-score | 1.03 | 0.79, | 1.35 |  | 0.95 | 0.72, | 1.25 |  | 0.97 | 0.72, | 1.29 |  | 0.99 | 0.74, | 1.32 |  |
| Practice organization | z-score | 1.15 | 0.89, | 1.48 |  | 1.07 | 0.80, | 1.42 |  | 1.14 | 0.87, | 1.49 |  | 1.05 | 0.78, | 1.43 |  |
| Resources | z-score | 0.80 | 0.58, | 1.10 |  | 0.65 | 0.46, | 0.93 |  | 0.67 | 0.47, | 0.96 |  | 0.79 | 0.56, | 1.11 |  |
| Leadership behavior | z-score | 1.10 | 0.82, | 1.48 |  | 0.99 | 0.71, | 1.39 |  | 1.04 | 0.75, | 1.44 |  | 1.00 | 0.70, | 1.42 |  |

Effort-reward imbalance questionnaire (ERI) or medical assistant (MA)-specific work stress questionnaire; ^a^ Associations between adverse working condition exposures and the outcome “overall important medical error”, model additionally adjusted for age and leadership position (Model II); ^b^ Model II additionally adjusted for vigor; ^c^ Model II additionally adjusted for dedication; ^d^ Model II additionally adjusted for self-rated health as potential mediator; ^e^ risk ratio (RR) and ^f^ 95% confidence intervals (95% CIs); ^g^ for collaboration Model II RR: significant p-value < 0.05.

Table A2b. Risk of being concerned to have made an important medical error since baseline for the follow up period combined by exposure to adverse psychosocial working conditions at baseline including the mediators (Poisson regression).

|  |  | **Summary measure of important medical errors** | | | | | | | | | | | | | | |
| --- | --- | --- | --- | --- | --- | --- | --- | --- | --- | --- | --- | --- | --- | --- | --- | --- |
| **Characteristic** |  | **Model II ^a^** | | |  | **Depression^b^** | | |  | **Anxiety^c^** | | |  | **Work satisfaction^d^** | | |
|  |  | **RR^e^** | **95% CI^f^** | |  | **RR** | **95% CI** | |  | **RR** | **95% CI** | |  | **RR** | **95% CI** | |
| ERI model |  |  |  | |  |  |  | |  |  |  | |  |  |  | |
| Effort | z-score | 0.98 | 0.77, | 1.25 |  | 0.89 | 0.69, | 1.14 |  | 0.88 | 0.68, | 1.13 |  | 1.00 | 0.78, | 1.30 |
| Reward | z-score | 0.86 | 0.66, | 1.12 |  | 1.01 | 0.76, | 1.34 |  | 1.02 | 0.74, | 1.39 |  | 0.80 | 0.58, | 1.10 |
| ERI | z-score | 1.06 | 0.82, | 1.36 |  | 0.89 | 0.66, | 1.20 |  | 0.89 | 0.65, | 1.23 |  | 1.13 | 0.85, | 1.51 |
| MA-specific instrument |  |  |  |  |  |  |  |  |  |  |  |  |  |  |  |  |
| Workload | z-score | 1.18 | 0.93, | 1.51 |  | 1.08 | 0.84, | 1.40 |  | 1.06 | 0.81, | 1.37 |  | 1.26 | 0.98, | 1.62 |
| Job control | z-score | 1.00 | 0.78, | 1.27 |  | 0.91 | 0.71, | 1.17 |  | 0.89 | 0.69, | 1.14 |  | 1.00 | 0.78, | 1.27 |
| **Collaboration^g^** | z-score | 1.26 | 1.00, | 1.57 |  | 1.09 | 0.83, | 1.42 |  | 1.06 | 0.80, | 1.41 |  | 1.36 | 1.05, | 1.76 |
| Gratification | z-score | 1.03 | 0.79, | 1.35 |  | 0.93 | 0.72, | 1.20 |  | 0.94 | 0.71, | 1.25 |  | 1.04 | 0.78, | 1.38 |
| Practice organization | z-score | 1.15 | 0.89, | 1.48 |  | 1.04 | 0.78, | 1.38 |  | 1.02 | 0.77, | 1.36 |  | 1.18 | 0.88, | 1.58 |
| Resources | z-score | 0.80 | 0.58, | 1.10 |  | 0.73 | 0.51, | 1.02 |  | 0.75 | 0.53, | 1.05 |  | 0.79 | 0.55, | 1.13 |
| Leadership behavior | z-score | 1.10 | 0.82, | 1.48 |  | 0.93 | 0.67, | 1.30 |  | 0.93 | 0.66, | 1.32 |  | 1.12 | 0.81, | 1.56 |

Effort-reward imbalance questionnaire (ERI) or medical assistant (MA)-specific work stress questionnaire; ^a^ Associations between adverse working condition exposures and the outcome “overall important medical error”, model additionally adjusted for age and leadership position (Model II); ^b^ Model II additionally adjusted for depression (PHQ2); ^c^ Model II additionally adjusted for anxiety (GAD2); ^d^ Model II additionally adjusted for work satisfaction (COPSOQ); ^e^ risk ratio (RR) and ^f^ 95% confidence intervals (95% CIs); ^g^ for collaboration Model II RR: significant p-value < 0.05.

Table A3. Risk of being concerned to have made an important medical error across the full follow up period (summary measure variable*) by exposure to adverse psychosocial working conditions (dichotomized) at baseline (Poisson regression).

|  |  | **Summary measure of important medical errors*** | | | | |  |
| --- | --- | --- | --- | --- | --- | --- | --- |
| **Characteristic** |  | **Model I^a^** | |  | **Model II^b^** | |  |
|  |  | **RR^c^** | **95% CI^d^** |  | **RR** | **95% CI** |  |
| ERI model |  |  |  |  |  |  |  |
| Effort | High vs low^e^ | 0.68 | 0.35, 1.34 |  | 0.76 | 0.38, 1.51 |  |
| Reward | High vs low | **0.41** | **0.20, 0.86** |  | **0.46** | **0.21, 0.98** |  |
| ERI ratio (>1.0 items vs rest) | High vs low | 1.42 | 0.71, 2.86 |  | 1.38 | 0.69, 2.76 |  |
| MA-specific instrument |  |  |  |  |  |  |  |
| Workload (high) | High vs low | 1.14 | 0.63, 2.05 |  | 1.18 | 0.66, 2.13 |  |
| Job control (low) | High vs low | 0.72 | 0.40, 1.31 |  | 0.84 | 0.46, 1.53 |  |
| Collaboration (poor) | High vs low | 1.64 | 0.94, 2.86 |  | 1.54 | 0.89, 2.67 |  |
| Gratification (low) | High vs low | 0.63 | 0.33, 1.18 |  | 0.55 | 0.29, 1.01 |  |
| Practice organization (poor) | High vs low | **1.77** | **1.02, 3.10** |  | 1.58 | 0.88, 2.83 |  |
| Resources (lack of) | High vs low | 0.75 | 0.38, 1.47 |  | 0.75 | 0.39, 1.46 |  |
| Leadership (poor behavior) | High vs low | 1.23 | 0.67, 2.26 |  | 1.15 | 0.63, 2.07 |  |

Effort-reward imbalance questionnaire (ERI) or medical assistant (MA)-specific work stress questionnaire; * perceived concerns about having made an important medical error reported for the last three months, 12 months and since baseline merged into a single variable (any affirmative response vs none); ^a^ unadjusted; ^b^ additionally adjusted for age and leadership position; ^c^ risk ratio (RR) and ^d^ 95% confidence intervals (95% CIs); ^e^ all exposure variables are dichotomized based on highest tertile vs remaining tertiles (high vs. low) except for the ERI-ratio; numbers in bold: significant p-value < 0.05.

Table A4. Risk of being concerned to have made an important medical error across the last 3 months at follow-up by exposure to adverse psychosocial working conditions at baseline (Poisson regression).

|  |  | **Important medical error last 3 month** | | | | |  |
| --- | --- | --- | --- | --- | --- | --- | --- |
| **Characteristic** |  | **Model I^a^** | |  | **Model II^b^** | |  |
|  |  | **RR^c^** | **95% CI^d^** |  | **RR** | **95% CI** |  |
| ERI model |  |  |  |  |  |  |  |
| Effort | z-score | 1.02 | 0.76, 1.38 |  | 1.06 | 0.78, 1.44 |  |
| Reward | z-score | 0.82 | 0.57, 1.17 |  | 0.84 | 0.59, 1.20 |  |
| ERI ratio | z-score | 1.08 | 0.80, 1.44 |  | 1.06 | 0.80, 1.42 |  |
| MA-specific instrument |  |  |  |  |  |  |  |
| Workload | z-score | 1.17 | 0.86, 1.59 |  | 1.19 | 0.87, 1.63 |  |
| Job control | z-score | 0.98 | 0.70, 1.38 |  | 1.02 | 0.72, 1.44 |  |
| Collaboration | z-score | 1.25 | 0.90, 1.74 |  | 1.24 | 0.90, 1.71 |  |
| Gratification | z-score | 1.16 | 0.79, 1.71 |  | 1.13 | 0.76, 1.66 |  |
| Practice organization | z-score | 1.15 | 0.79, 1.66 |  | 1.12 | 0.76, 1.64 |  |
| Resources | z-score | 0.90 | 0.58, 1.38 |  | 0.89 | 0.58, 1.38 |  |
| Leadership behavior | z-score | 1.06 | 0.72, 1.56 |  | 1.04 | 0.72, 1.52 |  |

Effort-reward imbalance questionnaire (ERI) or medical assistant (MA)-specific work stress questionnaire; ^a^ unadjusted; ^b^ additionally adjusted for age and leadership position; ^c^ risk ratio (RR) and ^d^ 95% confidence intervals (95% CIs).

Table A5. Risk of being concerned to have made an important medical error across the last 12 months at follow-up by exposure to adverse psychosocial working conditions at baseline (Poisson regression).

|  |  | **Important medical error last 12 month** | | | | |  |
| --- | --- | --- | --- | --- | --- | --- | --- |
| **Characteristic** |  | **Model I^a^** | |  | **Model II^b^** | |  |
|  |  | **RR^c^** | **95% CI^d^** |  | **RR** | **95% CI** |  |
| ERI model |  |  |  |  |  |  |  |
| Effort | z-score | 0.92 | 0.66, 1.29 |  | 0.98 | 0.70, 1.37 |  |
| Reward | z-score | **0.66** | **0.49, 0.89** |  | **0.70** | **0.51, 0.97** |  |
| ERI ratio | z-score | 1.18 | 0.87, 1.60 |  | 1.15 | 0.84, 1.56 |  |
| MA-specific instrument |  |  |  |  |  |  |  |
| Workload | z-score | 1.18 | 0.87, 1.61 |  | 1.22 | 0.90, 1.65 |  |
| Job control | z-score | 0.92 | 0.67, 1.27 |  | 1.00 | 0.71, 1.39 |  |
| Collaboration | z-score | 1.32 | 0.98, 1.76 |  | 1.26 | 0.93, 1.70 |  |
| Gratification | z-score | **1.48** | **1.04, 2.09** |  | 1.39 | 0.98, 1.98 |  |
| Practice organization | z-score | 1.18 | 0.89, 1.58 |  | 1.10 | 0.82, 1.48 |  |
| Resources | z-score | 0.88 | 0.58, 1.34 |  | 0.87 | 0.58, 1.31 |  |
| Leadership behavior | z-score | 1.20 | 0.84, 1.69 |  | 1.14 | 0.80, 1.62 |  |

Effort-reward imbalance questionnaire (ERI) or medical assistant (MA)-specific work stress questionnaire; ^a^ unadjusted; ^b^ additionally adjusted for age and leadership position; ^c^ risk ratio (RR) and ^d^ 95% confidence intervals (95% CIs); numbers in bold: significant p-value < 0.05.

Table A6. Risk of being concerned to have made an important medical error since baseline by exposure to adverse psychosocial working conditions at baseline (Poisson regression).

|  |  | **Important medical error since baseline** | | | | |  |
| --- | --- | --- | --- | --- | --- | --- | --- |
| **Characteristic** |  | **Model I^a^** | |  | **Model II^b^** | |  |
|  |  | **RR^c^** | **95% CI^d^** |  | **RR** | **95% CI** |  |
| ERI model |  |  |  |  |  |  |  |
| Effort | z-score | 1.05 | 0.76, 1.43 |  | 1.12 | 0.82, 1.52 |  |
| Reward | z-score | 0.82 | 0.61, 1.11 |  | 0.91 | 0.66, 1.26 |  |
| ERI ratio | z-score | 1.14 | 0.85, 1.52 |  | 1.09 | 0.81, 1.47 |  |
| MA-specific instrument |  |  |  |  |  |  |  |
| Workload | z-score | 1.30 | 0.94, 1.81 |  | 1.36 | 0.98, 1.88 |  |
| Job control | z-score | 1.03 | 0.75, 1.41 |  | 1.15 | 0.82, 1.60 |  |
| Collaboration | z-score | **1.35** | **1.06, 1.73** |  | 1.27 | 1.00, 1.63 |  |
| Gratification | z-score | 1.10 | 0.80, 1.51 |  | 0.99 | 0.71, 1.39 |  |
| Practice Organization | z-score | 1.29 | 0.98, 1.69 |  | 1.19 | 0.89, 1.58 |  |
| Resources | z-score | **0.61** | **0.40, 0.95** |  | **0.60** | **0.39, 0.92** |  |
| Leadership behavior | z-score | 1.16 | 0.82, 1.64 |  | 1.10 | 0.76, 1.57 |  |

Effort-reward imbalance questionnaire (ERI) or medical assistant (MA)-specific work stress questionnaire; ^a^ unadjusted; ^b^ additionally adjusted for age and leadership position; ^c^ risk ratio (RR) and ^d^ 95% confidence intervals (95% CIs); numbers in bold: significant p-value < 0.05.
